# Partnership for health promotion as a platform to improve public health outcomes in Small Island Developing States: a case study of the Union of the Comoros

**DOI:** 10.3389/fpubh.2025.1666667

**Published:** 2025-11-14

**Authors:** Andrea Luciani, Sandrine Abega, Abdoulaye Diarra, Pamela Drameh, Matshidiso Moeti

**Affiliations:** 1 World Health Organization, Regional Office for Africa, Brazzaville, Republic of Congo; 2 World Health Organization, WHO Country Office in Central African Republic, Bangui, Central African Republic; 3 World Health Organization, WHO Country Office in the Union of the Comoros, Moroni, Comoros

**Keywords:** civil society organizations (CSOs), small island developing states (SIDS), community-based health interventions, health promotion, universal health coverage (UHC), Comoros

## Abstract

In the Comoros, the World Health Organization (WHO) initiated a partnership with Civil Society Organizations (CSOs) aimed at improving health outcomes of target population. Health improvements were associated with increased service reach and selected observed changes in service indicators, reaching over 30,000 individuals (1), including women and children in underserved areas. Specific outcomes include strengthened access to selected maternal, neonatal and child health services; expanded community outreach and early detection activities relevant to noncommunicable conditions, such as oral health, women’s cancers, hypertension, diabetes, hepatitis. The collaborative approach also strengthened the capacity of CSOs to deliver health services. The WHO-CSO partnership in the Comoros demonstrates the potential of strategic collaborations to enhance health outcomes in resource-limited settings. The findings support the scalability of such initiatives across similar contexts in Africa and other small island developing states (SIDS).

## Highlights

*What is already known about this topic*

- Role of Civil Society Organizations (CSOs): CSOs are recognized for their ability to reach underserved populations, promote health advocacy, and strengthen healthcare delivery in low-resource settings. They play a crucial role in implementing community-based health initiatives, particularly in resource-constrained environments like small island developing states (SIDS).
- Partnerships for Health Promotion: Collaborative efforts between international organizations like WHO and local CSOs have demonstrated effectiveness in improving health outcomes. Such partnerships are essential in achieving scalable, sustainable health solutions in low-resource environments, where government capacity may be limited.

*What this study adds*

- The practice highlights the tangible health improvements achieved through the WHO-CSO partnership in the Comoros, such as reducing dental caries in children, enhancing cancer screening, and improving maternal health services, reaching over 30,000 individuals.
- The practice provides evidence that strategic collaborations between WHO and local CSOs not only accelerate the improvement of immediate health outcomes but also enhance the capacity of CSOs to sustain and scale health interventions, offering a model for similar initiatives in other SIDS and low-resource settings.

*How this study might affect research, practice, or policy*

- This practice offers a framework for policymakers to design effective health interventions in resource-limited settings, particularly by leveraging partnerships with Civil Society Organizations (CSOs) to improve health outcomes and address healthcare challenges in underserved communities by making them actors and final beneficiaries.”

## Introduction

The Union of the Comoros, comprising three islands in the Indian Ocean—Grande Comore, Moheli, and Anjouan-is a small island developing state (SIDS) with a population of approximately 850,000. The country faces significant health challenges, including high rates of maternal, neonatal, and child mortality, a growing burden of non-communicable diseases (NCDs) (1–4), and limited access to healthcare services, particularly in remote areas. The World Health Organization (WHO)'s “Triple Billion” goals, which aim to ensure 1 billion more people benefit from universal health coverage (UHC), 1 billion more people are protected from health emergencies, and 1 billion more people enjoy better health and wellbeing (5), have guided health initiatives in the Comoros. However, achieving these goals in SIDS like the Comoros requires tailored approaches that address the unique context and challenges faced by these countries (6, 7).

In response to these challenges, WHO Comoros initiated a partnership with six CSOs to implement health promotion projects. These projects targeted key areas, including reducing maternal, neonatal, and child mortality; combating NCDs; and increasing community involvement in preventing and responding to public health emergencies.

In this practice article, using both qualitative and quantitative methods, we review and discuss the observed changes brought about by these partnerships on health outcomes in the Comoros, with a focus on understanding how collaborative efforts can address the specific needs of SIDS.

## The WHO-civil society initiative for the promotion of health in the union of Comoros

The partnership between WHO, Ministry of Health and CSOs in Comoros was created against the backdrop of the need for community engagement and participation in health programs. The first cohort of this initiative was rolled out in 2022, with a partnership between four CSO for the implementation of health projects contributing to the reduction of maternal, neonatal, and infant mortality at community level and the reduction of communicable and non-communicable diseases from April to November 2022. Based on the successes achieved in the first cohort, WHO, in collaboration with the Ministry of Health scaled up the initiative to be replicated throughout the Comoros, by increasing the budget allocated to CSOs and extending the implementation period to 7 months. In 2023, five CSOs were partnered to carry out various community projects from June to December 2023, with major focus on awareness campaigns targeting 3,000 women and 5,000 children.

The priority areas covered by the CSOs were:

1. Implementation of actions to reduce maternal and child mortality rates through improved access to healthcare and education which was implemented by Caritas Comores;
2. Development and implementation of interventions to prevent and control non-communicable diseases, with a focus on diseases such as cancer, hepatitis, oral diseases, hypertension and diabetes. These were implemented by Association Comorienne contre les Cancers chez la Femme (ACCF), Action pour le Développement Durable et l’Environnement (ADDE), Collaboration Action Pérennisation (CAP) and Association (SITARA);
3. Implementation of interventions to promote healthy diets which was carried out by the Fédération des Acteurs pour le Développement Economique et Social de l’Île de Mohéli (FADESIM).

The initiative involved a multi-phase approach (1), including:

1. *Needs Assessment throughout the Comoros, identification of CSOs and selection of CSOs projects*: A comprehensive assessment was conducted 3 months before start of implementation of activities, to identify the specific health challenges in the Comoros. It was the responsibility of the Ministry of Health to carry out a preliminary identification of CSOs eligible for the initiative within an existing database of active CSOs in the country, capable of making significant impact in the communities. This initial phase was conducted meticulously to guarantee inclusivity and relevance to ensure that the shortlisted organizations align with the program’s objectives. Following this initial identification, the Ministry proceeded with the collection of official documents from the shortlisted CSOs. These documents were essential for assessing the legitimacy and operational capacity of the organizations. The process involved gathering comprehensive documentation to provide a clear understanding of each organization’s structure, mission and relevance to the initiative. The collected documents underwent a thorough verification process carried out by the External Relations and Partnerships teams of the WHO, including both the WHO Country Office in Comoros and the WHO Regional Office for Africa. This verification adhered strictly to the principle of due diligence outlined in the Framework of Engagement with Non-State Actors (WHO FENSA) (8). By applying these principles, the WHO ensured that all partnerships would be built on transparency, accountability and alignment with its global health’s mandate.
2. *Capacity Development*: Preselected CSOs were provided with training and resources to enhance their organizational capacity but also to empower them with the knowledge and skills necessary to develop quality project proposals, implementation plan, and a detailed budget. This training was provided by WHO experts from the country office and the regional office, with support from the United Nations System Coordination Office. This implied the knowledge of the Partnership cycle, along with the WHO FENSA.
3. *Collaborative Projects*: WHO, in collaboration with the Ministry of Health in Comoros, launched a nation-wide call for submission of projects for the promotion of health. The submitted projects went through an extensive selection process. At this stage and according to the WHO FENSA, a risk assessment process was performed to authorized legally binding collaboration with the CSOs (6). The selected projects activities started on April 1, 2022, to close November 31, 2022. The same was applied for the second cohort of the initiative under which projects started in June 2023 and will close in December 2023.
4. *Project implementation and final evaluation*: The WHO office has carried out several supervision missions to monitor the progress of CSOs. These missions enabled the difficulties encountered to be identified and pragmatic solutions found. At the end of the Initiative, a final evaluation took place to assess the impact achieved, document the results, and valorize the achievements of the projects.

## Sampling and intervention

Based on contextual determinants specific to a Small Island Developing State (SIDs), the prioritization matrix of targeted population utilized five key criteria: burden of morbidity and risk, service coverage gaps, operational feasibility, equity/access, and systemic impact potential, with at least four dimensions applying to those in view of enabling measurable short-term gains while reinforcing public service delivery.

The first group consisted of children aged 3–10 years identified through the channel of disadvantaged schools. This demographic faces a high prevalence of untreated dental caries, a preventable condition with low-cost interventions. The absence of structured preventive services such as fluoridation and hygiene education further underscores the need for targeted action. Leveraging the school environment as a universal channel ensures stable cohorts and streamlined follow-up through collaboration with teachers, parents and local administrative authorities.

**Figure 1:**
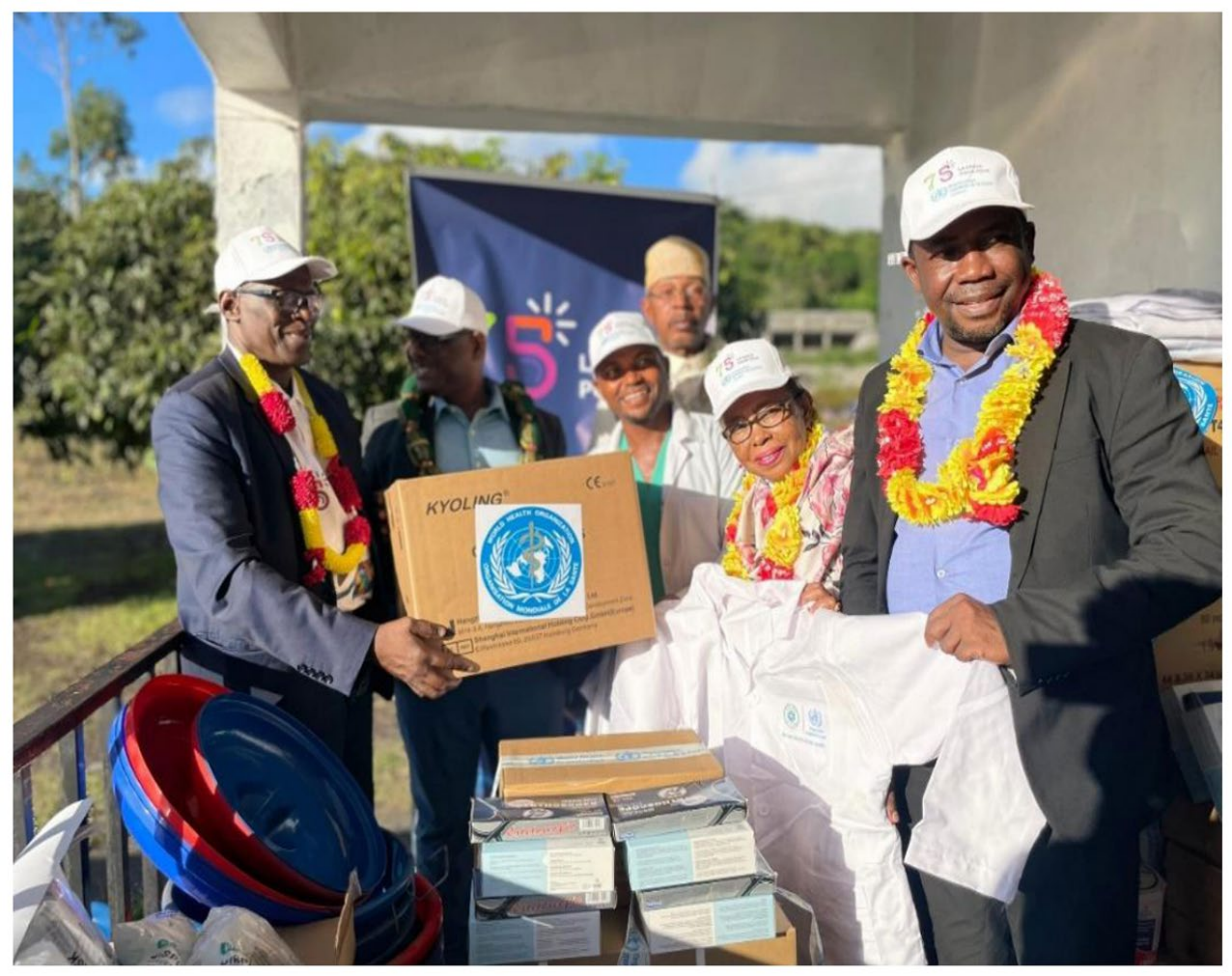
Handover of health equipment and kits in Domoni (Ajouan island), With Dr. DIARRA, Abdoulaye, WHO Representative to the Union of the Comoros, the Minister of Health, the town authorities and village associations.

The second group prioritized women facing financial and geographic barriers in accessing essential preventive care of breast and cervical cancers (e.g., HPV testing, imaging). By integrating these services into existing healthcare structures such as midwifery networks and strengthening referral pathways, the intervention improved accessibility while fostering informed demand through targeted information, education, and communication efforts (Figure 1).

**Figure 2:**
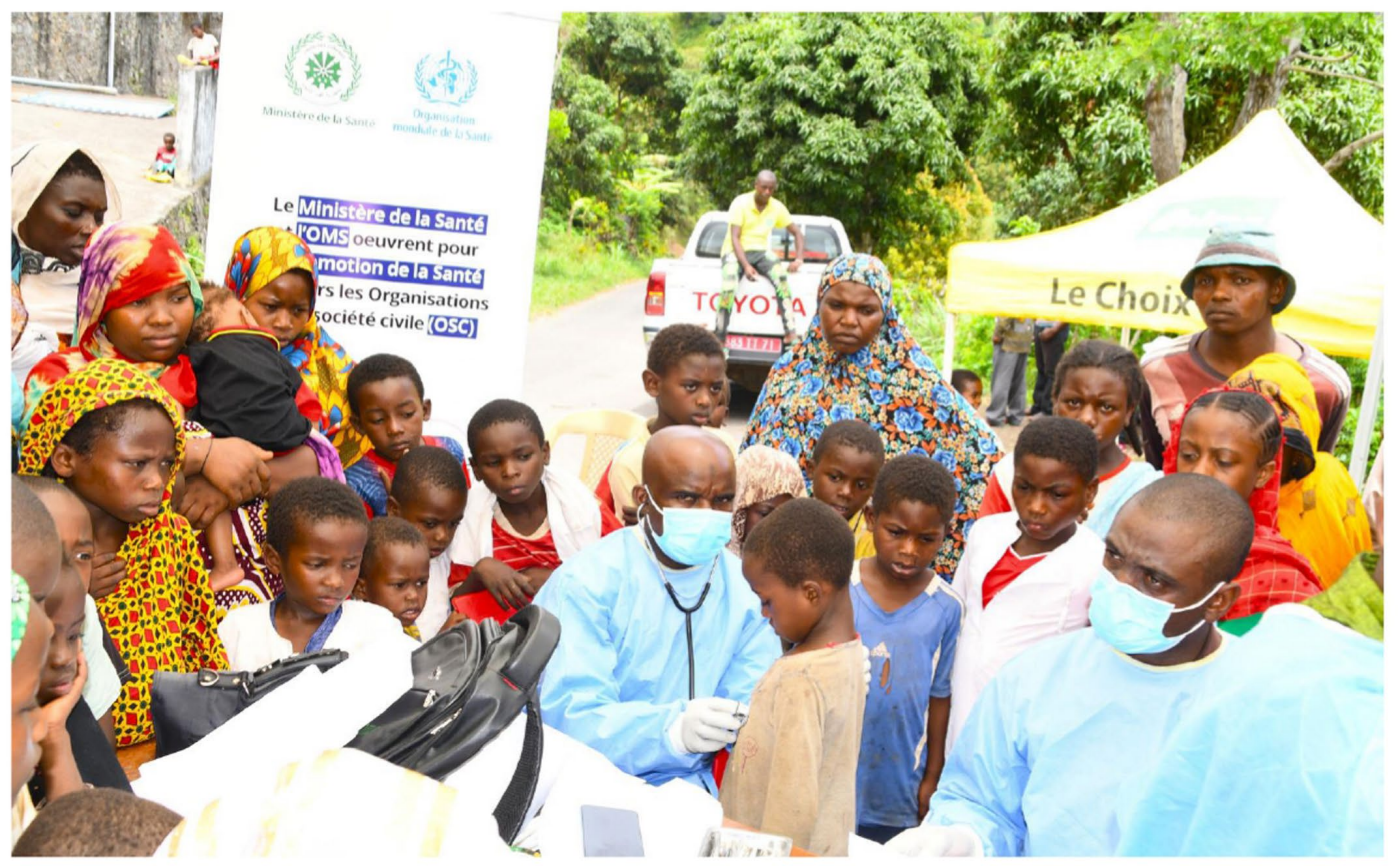
Medical consultation of Koni children by the medical team mobilized by ADDE, as part of the implementation of the “Project to Improve Oral Health among Students in Public Primary Schools in Koni” (Anjouan Island).

Finally, maternal, neonatal, and infant health interventions targeted pregnant women, women of reproductive age, infants, and rural communities with limited healthcare access. Deploying mobile health units equipped with solar-powered solutions ensured continuity in antenatal care, vaccinations, and essential diagnostics. Strengthening community networks further enhanced service delivery in remote areas, directly addressing geographic inequities (Figure 2).

## Health outcomes of the partnerships

The WHO-CSO partnership reached over 30,000 individuals over the 2 years the WHO-CSOs partnerships were implemented across the three islands through the provision of health and consultation services, capacity development activities and awareness raising interventions by utilizing efficient delivery channels such as schools, universities, midwifery platforms, and mobile teams. In doing so, they generate systemic benefits—building local capacities, reinforcing referral pathways, and creating sustainable service models—that support long-term scalability to broader populations. These interventions focused on some of the most pressing health challenges in the country, with notable successes in improving access to essential health services. Beneficiary identification relied on data collection methods, including primary registers maintained by CSOs during their interventions. These records encompassed attendance sheets, clinical registers for consultations, screenings, vaccinations, dental care, imaging services, and referral logs. To ensure the accuracy and reliability of the data provided, WHO Comoros country Office and Regional Office for Africa and the Ministry of Health conducted desk reviews and on-site verifications during joint supervision efforts (Figure 1).

### Reduction in dental caries

- The Action pour le Développement Durable et l’Environnement (ADDE) focused on improving oral health among schoolchildren in the vulnerable neighborhoods of Koni, Chiwé Sangani and Chitsangani, as well as in rural areas of Anjouan. Through awareness campaigns, free dental treatments, and the distribution of health kits, the 1,839 schoolchildren were consulted (1,019 girls and 820 boys). Over a period of 2 years of the initiative, ADDE recorded a reduction of dental caries up to 55% in certain classes. This outcome was particularly observable given the limited access to dental care in these communities, where untreated dental caries are common.

### Enhanced cancer screening and prevention

- The Association Comorienne contre les Cancers chez la Femme (ACCF) targeted the prevention and early detection of breast and cervical cancers, two leading causes of morbidity and mortality among women in the Comoros. The project reached 3,548 individuals through awareness campaigns, and 673 mammograms and 131 cervical screenings were conducted, leading to early diagnosis and treatment of cases that might have otherwise gone undetected. The initiative also facilitated the timely medical evacuation of patients to Mauritius for advanced cancer care, highlighting the importance of cross-border partnerships in addressing healthcare needs that cannot be met locally.

The table below shows an example of the results of the community project led by the Comoros Association to fight Women’s Cancer (ACCF) (Table 1).

**Table 1:** Key information and results of the project implemented by the Comoros Association to Fight Women’s Cancer (ACCF) under the Breast and Cervical Cancer prevention initiative (2022–2023).

Partner NGO	Comoros association to fight women’s cancer (ACCF)
Title of the project	Scaling up breast and cervical cancer prevention activities in the Union of the Comoros
Target areas	Ndzuwani, Mwali, Ngazidja
Implementation timeline	2022–2023
Grant amount	50,000 USD (about 22,000,000 KMF)
Objectives of the project	Establishment of a mechanism for access to early detection of breast and cervical cancers. Improving access to clinical cancer care by facilitating the movement of poor patients to Mauritius Awareness campaigns and training
Key Results	3,548 people reached, including 2,613 women and 935 men on the three islands, by awareness-raising talks on risk actors and the importance of early detection of breast and cervical cancers thanks to 2,600 leaflets produced to raise awareness of early detection production of a short film to raise awareness of cancer and broadcast on national TV and radio stations; 50,000 people reached through social media; 69 people trained in gynecologic all cancers (ACCF members and partners), including 66 women and 3 men, have, communication techniques and volunteering; 673 mammograms performed; 131 cervical smear screenings; 40 gynecological consultations provided; 33 mammograms and 46 breast ultrasounds; 03 patients evacuated to Mauritius;

### Improvement in maternal and child health

- The project provided healthcare services to 2,351 individuals, including prenatal care, vaccinations, and health education. The introduction of ultrasound services at the Salimani Hamahamet health center was a critical achievement, enabling early detection of complications in pregnancy and improving outcomes for both mothers and infants. This service, coupled with the mobile health teams that reached remote areas, expanded availability and uptake of selected maternal and neonatal services in the targeted regions.

### Prevention and control of NCDS

- The Fédération des Acteurs pour le Développement Économique et Social de l’Île de Mohéli (FADESIM) and Collaboration Action Pérennisation (CAP) addressed the growing burden of NCDs in the Comoros (2). These initiatives focused on screening for hypertension and diabetes, raising awareness about the dangers of psychotropic substances, and promoting healthy lifestyles. A total of 1,484 individuals were screened for hypertension and diabetes, and behavior change campaigns were conducted to encourage healthier living. The projects also included the distribution of blood pressure and blood sugar monitors, which empowered individuals to manage their health more effectively.

### Prevention of viral hepatitis

- Association SITARA screened 1,482 people for HBV. 734 of these were men and 748 were women. A total of 54 positive cases of HBV were detected, corresponding to a prevalence of 3.64%. 215 people, of whom 105 were men and 110 were women, were vaccinated against HBV;

### Capacity building and sustainability

One of the key outcomes of the WHO-CSO partnership was the notable enhancement of the capacity of CSOs to deliver health services. WHO provided training and resources to CSOs, focusing on project management, health service delivery, and community engagement. This capacity-building effort ensured that CSOs were equipped to implement and sustain health interventions effectively, even in the face of challenges such as resource constraints and cultural barriers.

The initiative’s success in the first phase led to increased funding for CSOs in the second phase, with extended timelines and larger grant amounts. For example, the grant amount for ACCF increased from 2022 to 2023, reflecting the project’s demonstrated impact and the need to scale up its activities. Similarly, ADDE received an increased grant to expand its oral health initiative to additional rural areas, further illustrating the scalability of these interventions.

The sustainability of the partnership was also evident in the strong engagement of community members, who played an active role in the implementation of health projects. This community involvement not only enhanced the relevance and acceptance of the interventions but also ensured that the benefits of the projects would be sustained beyond the project period.

## Key lessons learned

In the face of the many challenges we face, a unified vision of coherent, coordinated and comprehensive responses from the multilateral system is more important than ever. The implementation of solutions requires action and participation from all sectors of society, including Governments at all levels, the private sector, academia, individuals, and civil society, in particular. CSOs have a unique position to represent and reach populations and help promote universal health coverage. CSOs often engage in advocacy efforts to raise awareness about health issues within the community and at the national CSOs may contribute to strengthening the capacity of healthcare systems by providing training and resources to healthcare professionals. This enhances the overall quality of healthcare services in the country. CSOs work closely with local communities to understand their specific health needs and challenges. They facilitate community-based initiatives, ensuring that healthcare interventions are culturally sensitive and tailored to local context (6).

The results of this practice underscore the added-value of WHO-CSO partnerships in addressing critical health challenges in the Comoros. The collaborative approach facilitated the delivery of targeted health interventions in underserved areas, leading to notable improvements in health outcomes. These findings align with existing literature, including the WHO Framework of engagement with non-State actors (WHA69.10) (8) on the role of CSOs in enhancing health service delivery in low-resource settings, where government capacity is often limited, and access to healthcare is constrained. The success of this can be attributed to several factors. First, the careful selection of CSOs with a strong presence in the community ensured that the interventions were culturally appropriate and well-received. Second, the capacity-building efforts by WHO equipped CSOs with the skills and resources needed to implement effective health projects, even in challenging environments. Third, the focus on sustainability, through both increased funding and community engagement, ensured that the benefits of the partnership would be long-lasting.

## Challenges and limitations

However, the practice also highlights some challenges that need to be addressed to enhance the effectiveness of such partnerships. On the one hand, resource constraints, particularly in the availability of funds and healthcare infrastructure, were a significant barrier to scaling up the interventions. Additionally, taking into consideration cultural sensitivity was crucial in tailoring health interventions to local contexts, requiring careful navigation to secure community acceptance and active participation. The experience in the Comoros underscores the importance of flexibility and adaptability in implementing health interventions in diverse cultural settings. On another hand, limitations were observed on the existence and availability of complete and accurate data (comparable pre-intervention dataset), specifically with regards to maternal and neonatal mortality. Consequently, the findings presented describe observed improvements with plausible and not strict attribution to the WHO-CSO partnerships.

## Sustainability

The findings of this practice have important implications for the broader application of WHO-CSO partnerships in other SIDS and low-resource settings. The success of the partnership in the Comoros suggests that similar approaches could be effective in addressing health challenges in other contexts with similar socio-economic and geographic characteristics. Moreover, the focus on capacity building and sustainability provides a model for how international organizations can work with local CSOs to achieve long-term health improvements.

## Conclusion

The WHO-CSO partnership in the Comoros serves as a model for enhancing health outcomes through collaborative efforts in resource-limited settings. The observable improvements in access to maternal, neonatal, and child health, as well as the scale up of the prevention and control of NCDs, demonstrate the potential of such partnerships to contribute to the WHO’s global health goals. The results suggest that structured WHO–CSO collaborations may foster tangible improvements in low-resource settings while building local capacities and potentially sustainable delivery models health outcomes (6). These observations should be interpreted with caution given the absence of complete pre-intervention data and a formal comparison design.

Future efforts should focus on scaling up these initiatives and exploring their applicability in other contexts where standardized measurement and complete comparators would be needed to assess effectiveness and maximize their impact. Given the success of the partnership for health in the Comoros, there is potential for replication in other SIDS and low-resource settings across Africa and beyond. By continuing to leverage the strengths of CSOs and fostering strategic collaborations, WHO and its partners can make significant strides toward achieving the “Triple Billion” goals and improving health outcomes for vulnerable populations worldwide.

## Data Availability

The original contributions presented in the study are included in the article/supplementary material, further inquiries can be directed to the corresponding author.
